# Genotyping Tools for *Mycobacterium ulcerans*-Drawbacks and Future Prospects

**DOI:** 10.4172/2161-1068.1000149

**Published:** 2014-05-05

**Authors:** Charles A Narh, Lydia Mosi, Charles Quaye, Samuel CK Tay, Bassirou Bonfoh, Dziedzom K de Souza

**Affiliations:** 1 Parasitology Department, Noguchi Memorial Institute for Medical Research, University of Ghana, Ghana; 2 Centre Suisse de Recherches Scientifiques en Côte d’Ivoire, Ivory Coast; 3 Biochemistry, Cell and Molecular Biology Department, University of Ghana; 4 Clinical Microbiology Department, School of Medical Sciences, Kwame Nkrumah University of Science and Technology, Ghana

## Abstract

*Mycobacterium ulcerans* infection (Buruli ulcer) is a neglected but treatable skin disease endemic in over 30 countries. *M. ulcerans* is an environmental mycobacteria with an elusive mode of transmission to humans. Ecological and Molecular epidemiological studies to identify reservoirs and transmission vectors are important for source tracking infections especially during outbreaks and elucidating transmission routes. Research efforts have therefore focused on genotyping strains of the mycobacteria from clinical and environmental samples. This review discusses genotyping tools for differentiating *M. ulcerans* strains from other environmental and Mycolactone Producing Mycobacteria (MPMs). We highlight tools that have been adapted from related fields and propose ways these could be enhanced to resolve intra-species variation for epidemiological, transmission, evolutionary studies, and detection of emerging drug resistant strains. In the wake of increasing cases of Buruli ulcer, cumulative efforts including improvement in diagnostic methods and fine-tuning of genotyping tools are crucial to complement public health efforts in reducing infections.

## Introduction

*Mycobacterium ulcerans* (MU) infection or Buruli ulcer (BU) is a necrotizing disease of the skin and soft tissues [1]. It is the third most common mycobacterial infection after tuberculosis (TB) and leprosy [2], and endemic in over thirty countries worldwide including Ghana, Togo, Benin, Côte d’Ivoire, Cameroon, Mexico, Papua New Guinea, South East Asia and Australia [3,4]. The mode of transmission is still not clear but emergence of BU cases has been associated with contact with slow moving water bodies and wet lands, in several epidemiological studies [5-7]. *M. ulcerans* has been detected in several aquatic habitats with its abundance directly correlated with increased disease burden in some BU endemic communities [8,9]. It is a slow grower in culture experiments, thus making it difficult to culture from aquatic environmental samples. Hence, majority of the efforts to understand the ecology, molecular epidemiology, distribution and transmission of MU strains have relied on molecular detection methods [10-15]. This review highlights existing methods for genotyping *M. ulcerans* in humans and environmental samples, and discusses molecular approaches to understanding transmission routes and other emerging issues. It focuses on genotyping tools for differentiating MU strains from other Mycolactone Producing Mycobacteria (MPMs) and proposes ways these tools, in combination with other finer typing techniques, could be adapted for epidemiological, transmission, evolutionary studies, and detection of emerging MU drug resistant strains.

## Micro-geographical distribution of *M. ulcerans*

*M. ulcerans* is an environmental mycobacteria [16]. Comparative genomic analyses suggest that *M. ulcerans* is probably evolving from a generalist-environmental bacterium to a niche-adapted specialist in the mammalian host [17]. Within an aquatic environment, *M. ulcerans* can be found at the air-water interface, form biofilms on surfaces and probably occupy microhabitats not directly exposed to light but aerated [18]. Like most environmental mycobacteria, *M. ulcerans* proliferates in aquatic environments with pH ranges 5.5 to 6.5 [19]. A few studies have suggested that *M. ulcerans* could be transmitted to susceptible hosts, via aerosols generated from activities in contaminated water bodies, inoculation via an open skin or even through drinking of water containing bacilli [20]. However, experimental field studies to test these hypotheses were not fine-tuned to adequately identify specific modes of transmission [16]. Such studies would have to rely on advances in environmental and molecular biology to identify habitats and reservoirs of *M. ulcerans* persistence and proliferation [8]. Polymerase Chain Reaction (PCR) positivity of aquatic samples to MU DNA has been much higher in biofilms compared to other environmental samples, with environmental strains detected being identical to clinical isolates within endemic areas [8,9]. This is quite important in tracing routes of transmission from specific aquatic environments to humans. That is, it is possible to identify specific risk environments by comparing DNA sequence similarity of isolates to those causing infections in humans. Also, in transmission studies, attempts to culture MU from environmental sources should therefore focus on methods that will concentrate MU [8,9].

## Diagnostic Methods

In MU infection, microscopy for acid fast bacilli (AFB) can be performed on tissue biopsies, excised tissues, swabs and Fine Needle Aspirates (FNA) [21-27]. Although staining procedures for AFB are inexpensive and less time consuming, they are of low sensitivity and specificity [28]. WHO therefore recommends a supplementary test; culture, PCR or histopathology for confirming BU cases [4]. Histopathological studies of excised tissue or biopsy specimen have provided insights into the necrosis of the soft tissue. These studies suggested that although bacilli load was relatively high within the central portions of wounds, there were significant numbers at the peripherals [23]. Thus, to prevent recurrence of infection, it is suggested that surgical procedures excise surrounding peripheral tissues in addition to the necrotic tissues [27]. It takes approximately 6-8 weeks to see visible colonies of MU in pure cultures [29]. Thus, culture cannot be solely relied on to confirm cases before commencing treatment [30]. Addition of biochemical and antibiotic susceptibility tests, on cultured isolates, would be seminal in evaluating efficacy of existing antimicrobials and reveal emerging drug resistance [30,31]. It is therefore important for both clinical and research laboratories to set up antimicrobial surveillance systems in addition to routine diagnosis.

Owing to the delay in culture results and insensitivity of microscopy, most diagnostic laboratories now rely on PCR or real-time PCR for prompt and accurate diagnosis [13]. However, the technique; equipment and reagents, are expensive and cannot be afforded by poorly resourced laboratories at point of care facilities [32-34]. An alternative to the latter is Loop-Mediated Isothermal Amplification (LAMP) which is a novel technique with reported superiority to PCR in diagnosing *M. ulcerans* infections [32,33,35]. It is a cost-effective and robust technique which has the potential to supplant PCR diagnosis of *M. ulcerans* infections in poorly resourced laboratories. However, further studies are needed to optimize it for routine clinical diagnosis.

## Signature sequences of Mycolactone Producing Mycobacteria

Mycolactone producing mycobacteria (MPM); *Mycobacterium pseudoshottsii*, *M. liflandii*, *M. xenopi*,* M. marinum* DL and *M. ulcerans* are of immense importance because of their pathogenicity in causing debilitating ulcers in both animals and humans [17,36-38]. MPMs are thought to have evolved from *M. marinum* by the acquisition of a plasmid, pMUM, which encodes different congeners of mycolactone (Figure 2), and acquisition of insertion sequences, including *IS2404* and *IS2606*, on both chromosome and plasmid [17,39]. Thus, *IS2404* and *IS2606* are genetic markers used to differentiate MPMs from *M. marinum* M [39,40]. Genetic comparisons of MPM plasmids, pMUM001 (*M. ulcerans*), pMUM002 (*M. liflandii*) and pMUM003 (*M. marinum* DL), revealed >98% sequence similarity [41]. Primers targeting genes coding the enoyl reductase (ER) and keto reductase (KR), enzymes involved in mycolactone synthesis (Figure 2), have been used in the detection of *M. ulcerans, M. liflandii* and *M. marinum* DL in environmental samples [9]. However, successful differentiation of MPMs relies on a combination of other genotyping tools (Table 1) and the use of polymorphic markers including tandem repeat loci (Table 2) for differentiating the three mentioned species [9,42].

## Genome summary and genetic makers for studying transmission of *M. ulcerans*

MU is thought to have recently evolved from *Mycobacterium marinum*, a fish pathogen, via horizontal transfer of 174 kbp plasmid and is patho-adapting to the mammalian host [17]. Sequence data have shown that the two species share >98% sequence homology, with MU undergoing significant genome reduction [17]. There is paucity of evolutionary data on MU but phylogenetic analyses of the different geographical strains have suggested two lineages. The recently evolved classical lineage, comprises strains from Africa, Australia and Southeast Asia, and the ancestral lineage include strains from Asia, Mexico and South America [43,44]. In one study, Ghanaian MU strains were suggested to have evolved from a Japanese strain, about 394 to 521 thousand years ago, with subtypes diverging recently [45]. Nonetheless, there is much genome similarity among all MU strains [17,39,46].

MU, Agy99, has 2 circular replicons, a 5,631,606-bp chromosome and a 174,155-bp plasmid, pMUM001 [41]. The pMUM001 plasmid (Figure 2) contains 4 copies of *IS2404* and 8 copies of *IS2606*, both used as genetic markers [9]. Also prominent are 81 coding domain sequences (CDS), of which, enoyl reductase (ER) and keto reductase (KR) genes, two key enzymes involved in biosynthesis of mycolacetone, are used as genetic markers as well [8,9,47]. It has an average G+C content of 62.5% [18]. The chromosome has 209 and 83 copies of *IS2404* and *IS2606* respectively and 4,281 CDS. There are numerous DNA sequences, within functional and non-functional genes, which have been employed to differentiate strains of *M. ulcerans* from other MPMs [14]. These include variable number tandem repeats (VNTRs) like locus 6, locus 19, locus 1, ST1, MIRU1 [34,48] and a few housekeeping genes for Multilocus Sequence Typing (MLST). Despite currently available tools for studying transmission, it is worth highlighting that these are still limited compared to malaria and tuberculosis studies for detecting emergence of resistant strains and carrying out molecular epidemiological studies [16].

## Ribotyping of 16S rRNA gene

The mycobacterial 16S rDNA, which encodes 16S rRNA, is of great evolutionary importance and has been widely used to trace phylogenies, differentiate strains of mycobacteria [49-51] and to resolve ambiguities in bacterial nomenclature [49]. The 16S rRNA gene is about 1.5 kbp and has both conserved and variable regions across different taxa [50]. Sequence comparison of this gene between *M. ulcerans* and *M. marinum* showed no significant difference with slight variation at the 3′-end, between MU strains [51]. Thus, the 16S rDNA has limited potential for use as a diagnostic marker for MU infections. Restriction Fragment Length Polymorphism (RFLP) of the amplified 3′-end however differentiated 29 MU isolates into three allelic profiles according to three geographical regions; Australia, Africa and Mexico [46]. It however, could not differentiate between MU and *M. marinum*, suggesting its poor adaptability as a tool for typing genetically related isolates within the same geographical area, particularly, in epidemiological studies where the specific organism is to be identified. An alternative to ribotyping, is the conserved *rpoB* gene, which encodes the beta subunit of RNA polymerase. It has been shown to have a higher discriminatory power than 16S rRNA typing [52].

## Pulse field gel electrophoresis (PFGE) and Amplified fragment length polymorphism (AFLP)

PFGE can be used to separate large fragments of DNA in an electric field, where the voltage switches in three directions. One centrally and two at 60°, on either sides of the gel. Following restriction digest, e.g. of a genomic DNA or plasmid, fragments can be run on a gel by PFGE and banding pattern compared [53]. This technique which may be used for genotyping or genetic fingerprinting has been used to compare *M. tuberculosis* isolates for epidemiological purposes [54]. Prior to the genome sequence of *M. ulcerans*, PFGE analysis of *M. marinum* and *M. ulcerans* showed a genome size difference of 200 kbp [55]. In AFLP, fragmented DNA is ligated to specific DNA sequences (adaptor). Primers complementary to the adaptor are then used to amplify these fragments. Gel electrophoresis of the amplicons then shows banding patterns useful for genetic variation studies [56]. AFLP study of three mycobacteria species classified 12 *M. ulcerans* isolates into two geographical types originating from Africa and Australia [57]. Following these findings, coupled with the high sequence similarity among MU isolates, there has not been much application of these tools in *M. ulcerans* genetic studies. Although these tools seem less attractive to BU researchers, they have been used in other fields for molecular epidemiological studies [54,56,58,59]. Similar approaches could be recommended to *M. ulcerans* research.

## IS*2404* and IS*2606* PCR and RFLP analyses

Sequence analyses of the *M. ulcerans* reference genome, Agy 99, have revealed that both IS*2404* and IS*2606* are present in multiple copies, on both chromosome and plasmid [41]. Subsequently, primers targeting these sequences have been designed for use in PCR detection of *M. ulcerans* in clinical, veterinary and environmental isolates [22,25,60,61]. Based on IS*2404* RFLP, Chemlal et al. [46] showed that six distinct genotypes of *M. ulcerans* could be differentiated corresponding to the different geographical origins; Africa, Australia, Mexico, Papua New Guinea, Japan and Suriname. In another study, a typing tool, IS*2426*-PCR, was develop to amplify the region between IS*2404* and another MU insertion sequence, IS*2606* [40]. Nine different IS*2426* PCR genotypes were observed. Furthermore, analysis of the banding pattern suggested genetic relatedness between MU isolates from Africa and Southeast Asia [40]. These findings intimate that IS*2404* and IS*2606* could be useful markers for differentiating geographical isolates and tracing evolutionary history of MU isolates. However, their discriminatory abilities for resolving subtle variation between isolates, within the same geographical region, need to be assessed. Furthermore, other MPMs have been shown to harbor copies of these sequences [39,62], suggesting that additional polymorphic markers are needed to differentiate these species. Therefore, during ecological studies to source track MU infection within endemic communities, it is imperative to combine different typing tools in differentiating MU from other MPMs. Thus, detection of IS*2404* and/or IS*2606* within environmental samples should be considered as presumptive detection of MU. Currently, IS*2404* positivity is sufficient to be considered as definite diagnosis for MU in BU cases, since there have been no reports of other MPMs causing infection in humans [4] except one recent study that suggested a propensity for *M. pseudoshottsii* to cause infection in humans [42]. Further studies are needed to substantiate the latter.

## Multilocus sequence typing (MLST)

In Multilocus sequence typing (MLST), sequences from different housekeeping genes are compared simultaneously [55]. MLST analysis, by Stinear et al. [55] using 8 housekeeping genes, on a panel of 18 *M. ulcerans* isolates from different geographical areas yielded six different genotypes, related to the six geographical areas of Surinam, Papua New Guinea, Mexico, Japan/China, Africa and Australia. This was consistent with findings by Chemlal et al. [46] using RFLP-IS*2404*. Further, comparative genomic studies of *M. ulcerans* and *M. marinum* genomes showed recent divergence of the former from *M. marinum*, by the acquisition of a plasmid [55]. While MLST could discriminate between different geographical isolates and possibly intra-species variation within the same geographical area, its practicability to routine PCR reactions in clinical diagnosis would be costly and time consuming. However, MU strain specific primers could be designed and optimized for use in a multiplex PCR reaction. Development of this tool would tremendously improve molecular epidemiological studies of MU.

## Variable number tandem repeats (VNTR-typing)

Variable number tandem repeats (VNTR) are locations in the genome where short sequences of DNA occur in a repetitive pattern, and adjacent to each other, “head-to-tail” [48,63]. These repeats which vary in number per genome can be used to differentiate between related species. In *M. ulcerans* and other MPMs, numerous VNTRs both within functional and non-functional genes have been identified at specific loci in the reference genome, Agy 99 [14,48]. PCR reactions targeting loci like, locus 6, locus 19, MIRU1 and ST1, all with variable repeats, have successfully been used to differentiate *M. ulcerans* from other MPMs [64,65]. It has also been used to resolve the apparent genetic homogeneity within and between geographical isolates [14]. A study in Ghana, by Hilty et al. [15] using a combination of two polymorphic VNTR loci, ST1 and MIRU1, on 72 African isolates, including 57 MU isolates from Ghana, revealed three different genotypes, with clonal clustering, suggesting genetic diversity of *M. ulcerans* in Ghana. In this study, the authors reported 3 strains of MU in the study area. One strain had a repeat of 2 for ST1 and 1 for MIRU1, genotype (2,1), and the others had genotypes (1,3) and (2,3) for ST1 and MIRU 1 (Table 2). All repeats were confirmed with sequencing, suggesting that length polymorphism (50 bp difference of PCR product, for each locus) has comparable discrimination power as sequence polymorphism, to reveal intra-species variation in MU. The addition of other polymorphic loci could therefore increase discrimination power, revealing more genotypes. This hypothesis was confirmed in a study, where the authors combined four VNTR markers (Table 2) to type environmental MU isolates [9]. MU isolates had VNTR profiles distinct from other MPMs (Table 2). Similarly, a recent study, but with cultured isolates, corroborated these findings [42]. In elucidating transmission routes, VNTR typing could be further improved, i.e. addition of other tandem repeat loci, to source-track MU infections to specific risk environments. Thus, it is possible to compare VNTR profiles of human isolates to environmental isolates, within endemic communities, to trace reservoirs and vectors of MU [9,42]. To increase efficiency of VNTR as a genotyping tool, a Multilocus VNTR analysis (MLVA) can be adapted and automated for typing MU strains. With this technique, PCR primers (in a multiplex PCR) for the different loci are labelled with different fluorescent dyes and products separated by capillary electrophoresis using an automated sequencer [63]. MLVA has become the reference typing method for *M. tuberculosis* [66].

## Single nucleotide polymorphisms (SNPs) and microarray analysis

**Figure 1:**
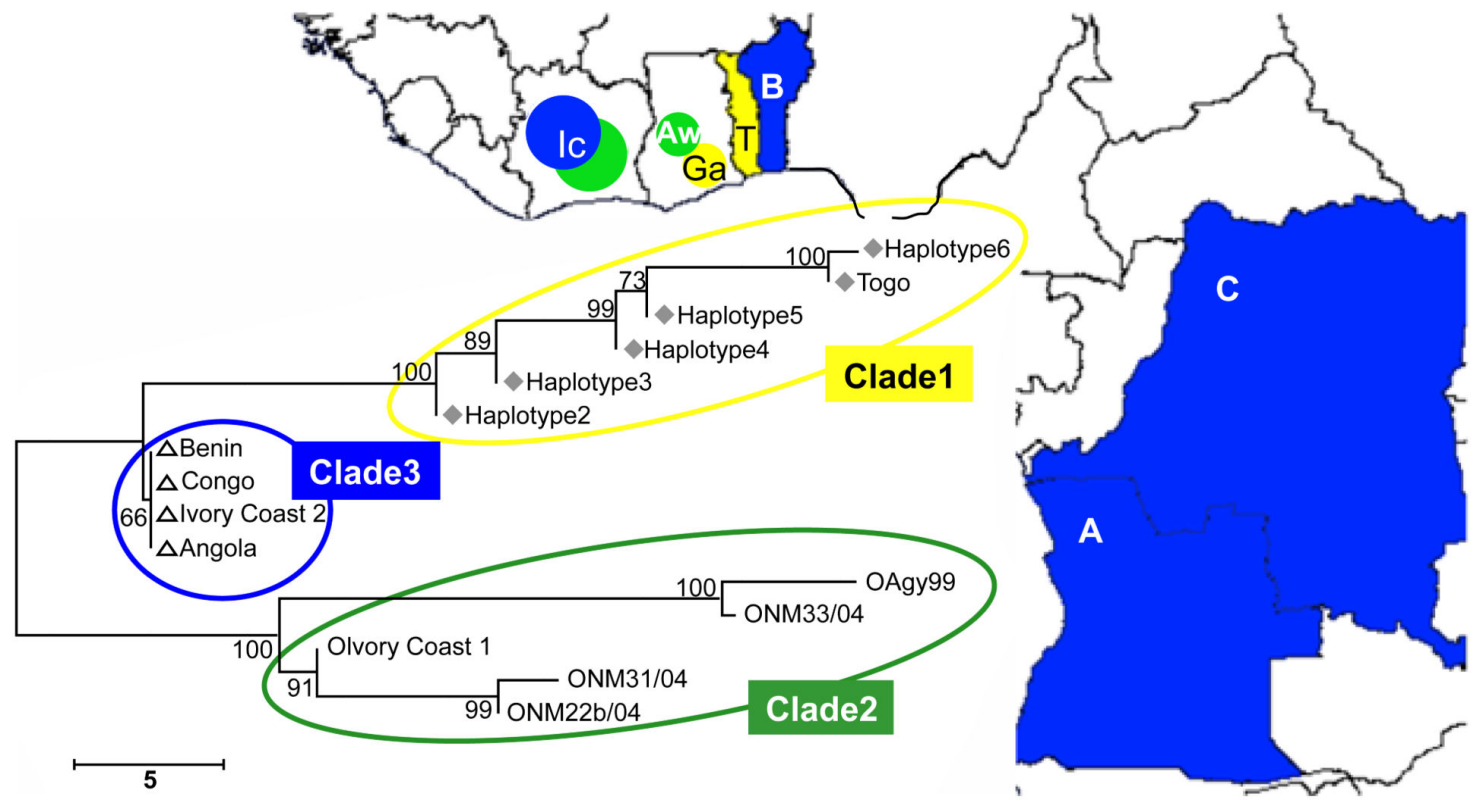
Geographical distribution of African *M. ulcerans* clades. Map of West-Africa, showing the distribution and SNP haplotypes of three African *M. ulcerans clades*. Clade 1: yellow; clade 2: green; clade 3: blue. AW: Amansie West; Ga: strains from the Densu river basin; IC: Ivory Coast; T: Togo; B: Benin; C: Democratic Republic of Congo; A: Angola. A neighbor-joining tree shows sub grouping of detected haplotypes from the Densu river basin together with the only strain from Togo into clade 1, strains from AW together with strain Agy99 and strain 1 from the Ivory Coast into clade 2 and all other strains from additional African countries into clade 3 (scale: number of differences at the SNP loci tested). From Roltgen *et al.* [34].

**Figure 2:**
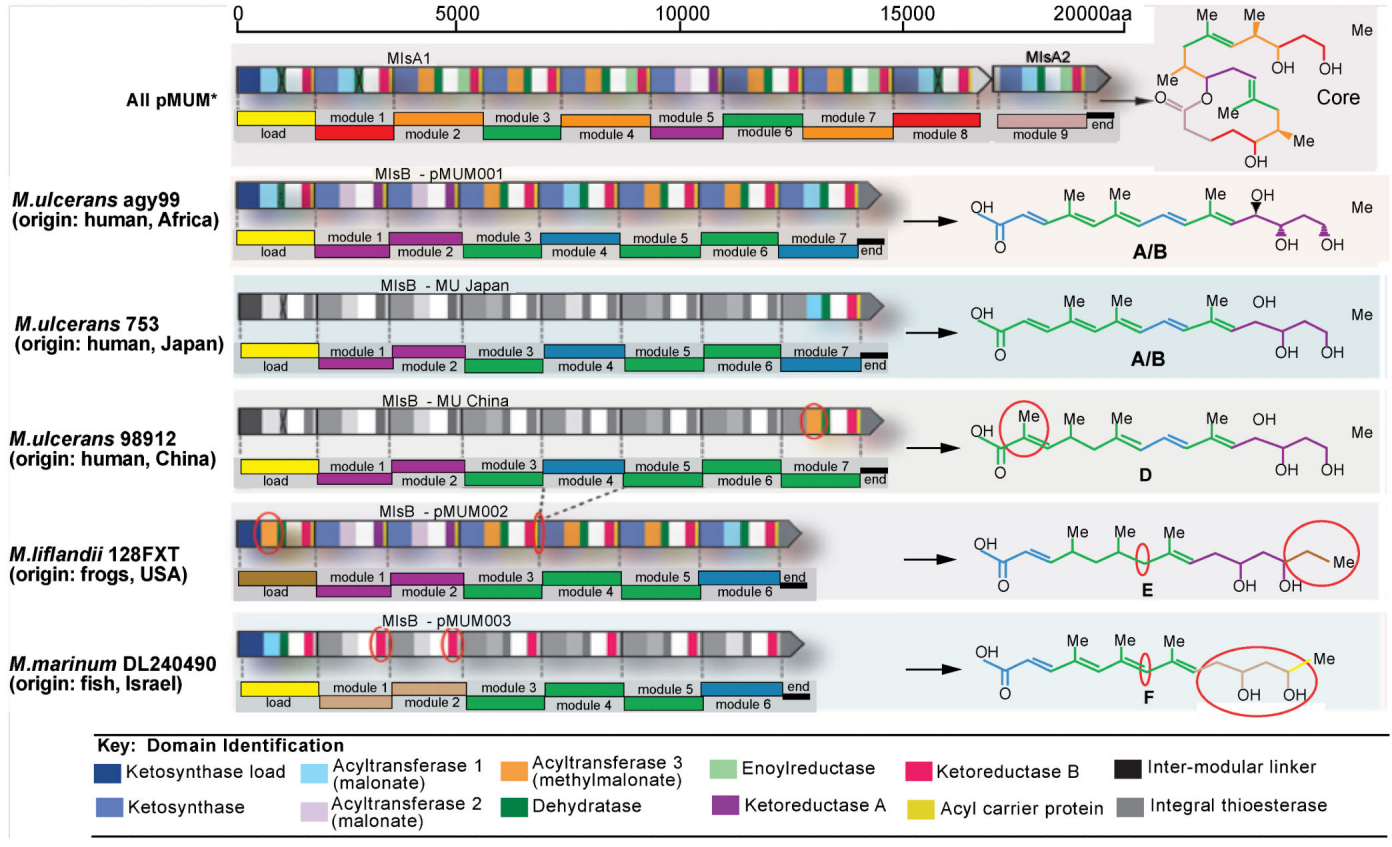
Genetic organization of the mycolactone biosynthetic cluster from pMUM001, pMUM002 and proposed organization for pMUM003. Mycolactone PKS module and domain structure is outlined, with the figure key showing the type of domains present in each of the modules. The mycolactone modules are colour coded based on the type of module responsible for the addition of each two-carbon unit. Shaded modules indicate that the DNA sequence of these regions is unknown or not yet confirmed. *Organisation of *mlsA1* and *mlsA2* for all pMUM examined to date, based upon toxin structures. From Pidot *et al*. [41].

Single Nucleotide Polymorphism (SNP typing), detects a single base pair mutation at a specific locus, revealing genetic variations between members of a species. Using this tool, various techniques from hybridization to enzyme-based methods and sequencing have been employed to reveal subtle differences in seemingly identical strains. Kaser et al. [67] employed SNP analysis of the IS*2404*, to genotype 83 *M. ulcerans* isolates from African countries. They identified 11 ISE-SNP types that differentiated regional strains into three haplotypes [67]. A similar study by the same group, using 94 protein coding genes, differentiated 54 Ghanaian MU isolates into 13 SNP haplotypes [45]. Following these findings, Roltgen et al. [34] developed a real-time PCR SNP typing method to genotype *M. ulcerans* patient isolates, collected from different parts of Ghana, and other isolates from Cote d’ Ivoire, Democratic Republic of Congo, Benin, Togo and Angola. Using 65 SNP loci, the authors identified nine haplotypes for the Ghanaian isolates. Neighbor-joining tree grouped isolates into three clades (Figure 1). Six haplotypes around the Densu River, southern Ghana, and isolates from Togo formed a clade. Isolates from central Ghana (three haplotypes) clustered with one Ivoirian isolate. The other African isolates formed the third clade. In one case, the authors observed dominance of one clonal complex and its clustering along the Densu River, suggesting possible focal transmission. Large scale application of SNP typing for epidemiological studies would involve the use of DNA microarrays on chips [68]. This technique has been used to differentiate *M. tuberculosis* from *M. bovis* BCG, through the identification of 18 regions of diversity (RD1-RD18) [69,70]. Although, this technique is costly, it is perhaps the most informative genotyping tool for detecting mutants in the population and picking up drug resistant strains. Reference laboratories could develop capacity in applying this tool to studying the population structure of MU, its evolutionary history and in monitoring the emergence of drug resistant strains.

## Modification of fine typing tools for *M. ulcerans* research

Advances in molecular biology, genomics, proteomics and bioinformatics have greatly accelerated research in understanding the molecular mechanisms of infection, pathology and treatment of various mycobacterial diseases, notably, TB and BU [2]. Sequencing of the genome of MU strain Agy99 has significantly improved studies on diagnosis and transmission [55]. However, these tools are currently inadequate in addressing critical research questions including the mode of transmission and hence the development of strategies for preventing and controlling the disease. A greater burden lies on molecular biologists and geneticists to develop finer typing tools for diagnosis and transmission studies. There is the need to rescan the published genomes of reference strains for nuanced sequences that could be used to differentiate between MU strains and other MPMs. Supplementary studies could explore the bacteria population structure within the different geographical areas, strain variation, and perceive how *M. ulcerans* may be evolving from an environmental niche to the human host. To achieve these, molecular epidemiological studies should not focus only on genotyping human MU isolates but need to also genotype environmental MU isolates. As previously discussed VNTR typing shows variation in environmental isolates [9] supporting SNP data that suggest clustering of a clonal complex within certain river basins [34]. Evidently, typing of more isolates, both human and environmental, would provide better insight into the bacteria population structure, which strains are niche adapting to the mammalian host, and the relative virulence of these strains, as inferred from mycolactone studies [71]. Recent VNTR typing of cultured MU isolates suggest that certain strains to be more readily cultured and/or transmissible than others [42]. Application of MLVA may increase the discriminatory power of VNTR typing. More specific primers targeting potential polymorphic markers, e.g. regions of difference (RD) [72], need to be designed to complement existing repertoire in diagnosis and for molecular epidemiological studies. Bioinformatics capacity and sequence data accessibility are strongly recommended to facilitate this. Additionally, high-throughput sequencing including next-generation sequencing and pyrosequencing, may improve quality of sequence data for SNP analysis [45]. RFLP-PCR would enhance strain differentiation if combined with capillary electrophoresis [63]. One important challenge however will be the standardization of genotyping protocols as most typing tools for MU give different genetic profiles. This could lead to varying designation of genotypes by different researchers and laboratories. We suggest that researchers develop and standardize genotyping protocols to facilitate easy identification and comparison of strains.

## Surveillance for emerging drug resistance

Rifampicin and streptomycin are the two WHO recommended antimicrobials that in combination are used for treating MU infections. Rifampicin inhibits bacteria DNA synthesis by inhibiting bacterial DNA-dependent RNA polymerase [73]. Streptomycin inhibits protein synthesis by binding to the bacterial 16S rRNA, interfering with the binding of formyl-methionyl-tRNA to the 30S subunit [74]. There have been reported cases of drug resistance to rifampicin in a mouse model [75] and in clinical isolates [76,77]. In the murine study, three MU mutants were isolated after rifampicin monotherapy. Molecular data showed that mutants harbored Ser416Phe and His420Tyr mutations in the *rpoB* gene [75]. Previous studies have shown that these mutations in the rpoB gene, confers rifampicin resistance to tubercle bacilli [73]. In another study, using sequence-based approach, a point mutation was detected in the *rpoB* gene at codon Ser522 leading to an amino acid change in 0.9% of the clinical isolates used [76]. Similarly, resistance to streptomycin has been reported in the closely related species, *M. tuberculosis*, in the *rpsL* gene [78]. These resistances could be compounded in cases where additional antibiotics are prescribed for the treatment of secondary infections, in ulcerative lesions [79].

These suggest that some MU strains have the propensity to develop resistance against these effective drugs. Current available tools for genotyping MU need to be improved and adapted for detecting resistant strains. SNP typing holds tremendous potential for this purpose. SNP typing been applied in various fields to detect drug resistant strains [76,78,80-83]. This technique in combination with VNTR typing could be used to determine strains that are becoming resistant. However, these techniques need to be tailored, obviating the need for expensive equipment, for use in developing countries, and at point of care facilities, where majority of cases are detected and treated. Additionally, MU isolates could be screened for susceptibility to existing antimicrobials, as complementary to the currently used ones or as alternatives, in the event of future resistance. New drugs with different modes of action could also be developed to combat future resistant strains [84].

## Conclusion

*M. ulcerans* infection is the third most common mycobacterial infections after tuberculosis and leprosy, however, it has received comparably less attention. Available tools for genotyping strains of the pathogen have shown low discriminatory power in resolving intra-species variation, particularly at regional levels. The application of finer genotyping tools including VNTR and SNP analysis, have shown heterogeneity within isolates from the same geographical area. Although these strains share nearly identical sequences, rescanning of the genome for nuanced sequences, refinement of existing tools and modification of tools from other fields, particularly TB, may help uncover other pathogenic subtypes. Concurrently, such advancements may further help detect emerging drug resistant strains. In the wake of increasing cases of Buruli ulcer, cumulative efforts including improvement in diagnostic methods and fine-tuning of genotyping tools are key to elucidating transmission routes, studying the molecular epidemiology of MU and detection of incipient resistant strains.

## Figures and Tables

**Table 1:** Summary of tools for genotyping *M. ulcerans*.

Tool	Markers	Strains	Polymorphism	Reference
Ribotyping	16S rRNA	3 strains (Australia, Africa and Mexico)	Low polymorphism for intra-species differentiation. 16S rRNA is conserved.	[46]
PFGE and AFLP	Whole genome	2 strains (Australia and Africa)	Limited by genetic homogeneity of MU strains, and restriction enzymes	[57]
RFLP	IS2404, IS2606 and polymorphic markers	6 strains (Africa, Australia, Mexico, Papua New Guinea, Japan and Suriname)	Low polymorphism for differentiating MU and MPM isolates from the same origin. Polymorphism depends on restriction enzymes used.	[40,46]
MLST	8 housekeeping genes	6 strains (Surinam, Papua New Guinea, Mexico, Japan/China, Africa and Australia)	Polymorphism may vary depending on gene loci	[55]
VNTR	Over 20 VNTR loci	8 genotypes (Ghana isolates) Variable for other geographical isolates	Differentiates strains (MU and MPMs) from the same origin. Genotypes depends on loci used.	[9,14,15,48,85]
SNP	ISE, 94 CDS	11 ISE-SNP types 13 SNP haplotypes	Strain specific differentiation	[45,67]

**Table 2:** VNTR profiles of MU and MPM strain genotypes from published data.

Published genotypes	VNTR Profiles				Reference
	MIRU1	Locus 6	ST1	Locus 19	
**M. ulcerans**					[9,42]
**A**	1	1	1	2	
**B**	3	1	1	2	
**C**	3	1	2	2	
**D**	1	1	2	2	
**MMDL**					
**E**	1	2	1	2	
**MLF**	1	2	2	1	
**MPS**	1	4	2	2	
**Amansie West, Ghana**					[15]
**MU strain 1**	1	ND	2	ND	
**MU strain 2**	3	ND	1	ND	
**MU strain 3**	3	ND	2	ND	
**Gh sequence MU strain**	ND	1	ND	2	[14]
**ITM 94-1324 Australian** **strain**	ND	1	ND	2	
**ITM 842 Surinam strain**	**ND**	1	ND	3	
**ITM 8756 Japanese** **strain**	**ND**	2	ND	4	

A, B, C, and D are *M. ulcerans* designated genotypes, and E is *M. marinum* DL. MMDL is *M. marinum* DL, MPS is *M. pseudoshottsii* and MLF is *M. liflandii*. ND, not done, Gh, Ghana. Work by Ablordey et al. [14] used 9 VNTR markers, excluding ST1 and MIRU1, but for the purpose of this review, only results for Loci 6 and 19 are shown.
